# Theta oscillations in prolactinomas: Neurocognitive deficits in executive controls

**DOI:** 10.1016/j.nicl.2020.102455

**Published:** 2020-09-30

**Authors:** Chenglong Cao, Wen Wen, Binbin Liu, Pan Ma, Sheng Li, Guozheng Xu, Jian Song

**Affiliations:** a The First School of Clinical Medicine, Southern Medical University, Guangzhou 510515, China; b Department of Neurosurgery, The General Hospital of Chinese PLA Central Theater Command, Wuhan 430070, China; c School of Psychological and Cognitive Sciences, Peking University, Beijing 100871, China; d Wuhan University of Science and Technology, Wuhan 430000, China; e Wuhan Children's Hospital, Tongji Medical College of Huazhong University of Science & Technology, China

**Keywords:** Prolactinomas, Theta oscillation, P300, N200, Response activation and inhibition

## Abstract

Impairment of cognitive functions has been reported in prolactinomas. However, the electrophysiological mechanisms of response activation and response inhibition in prolactinomas remain unclear. We recorded participants’ scalp electroencephalography (EEG) in a visual Go/Nogo task. Compared to the healthy controls (HCs), the patients demonstrated worse performance and their prolactin (PRL) levels negatively correlated with behavioral results. Meanwhile, patients’ P300 amplitudes in the Go and Nogo conditions were smaller than the HCs. The amplitudes of N200nogo in patients were smaller than the HCs as well. Lower frontal theta power was found in the patients than the HCs in both Go and Nogo conditions, which indicated a deficit in response activation and inhibition. Moreover, the PRL levels mediated the relationship between frontal theta power and behavior performance, implying that lower frontal theta power caused the dysfunction of response control by abnormally high PRL levels. Patients also showed lower occipital alpha power than the HCs, which suggested that the impaired response inhibition may arise from deficient attention control. Taken together, the present study revealed the neurocognitive discrepancies between prolactinomas and the HCs. The frontal theta oscillation was highlighted as the electrophysiological markers of the impaired response control in prolactinomas.

## Highlights

• Prolactin levels negatively correlated with prolactinomas’ executive controls.
• Prolactin levels mediated the correlation between frontal theta activity and inhibition control ability.
• Prolactinomas exhibited decreased frontal theta power in Go/Nogo task.
• The frontal theta oscillation was highlighted as the electrophysiological markers of the impaired inhibitory control in prolactinomas.

## 1 Introduction

Pituitary tumors are the most common intracranial tumors following the meningioma, accounting for about 16.5% of central nervous system tumors (Ostrom et al., 2018). Among all subtypes of pituitary tumors, prolactinomas are the most frequently reported and are commonly characterized by hypersecretion of prolactin (PRL) in the circulating blood (Fleseriu et al., 2006). Beyond the clinical symptoms caused by the tumor per se (Pal et al., 2019), there are emerging reports on cognitive impairments and emotional disorders (Pertichetti et al., 2019). Specifically, substantial evidence has shown that pituitary patients suffer from deficits in attention (Bala et al., 2016), working memory (Brummelman et al., 2011), emotion processing(Song et al., 2018), depression (Alcalar et al., 2013), and particularly executive function (Cao et al., 2017, Tooze et al., 2009).

Müssig et al. (2011) found that patients with pituitary adenomas showed greater impairments in executive function than those with other chronic illnesses. This suggested that it was the brain lesions, rather than general factors associated with chronic illness, that should be responsible for the deficit. Structural magnetic resonance imaging studies revealed that prolactinomas manifested decreased gray matter volume (GMV) in the prefrontal cortex (Yao et al., 2018) and the decreased GMV was correlated with the inhibition mechanism (Rubia et al., 2001). Besides impairing brain structures, PRL hypersecretion also affected the executive function and the influence may be indirectly mediated by the dopamine (Fitzgerald and Dinan, 2008). Dopamine neurotransmitters generally pass through hypophyseal portal blood from the hypothalamus to regulate the prolactin production and abnormally high PRL suppresses the secretion of dopamine (Ben-Jonathan and Hnasko, 2001). The altered balance between dopamine and prolactin levels may lead to cognitive impairments (Nieoullon, 2002). So far, few studies have investigated how PRL hypersecretion influences the evoked ERPs and neural oscillations associated with the response control.

N200 and P300 are widely used for the assessment of response control in Go/ Nogo tasks (Detandt et al., 2017, Dong et al., 2010, Huster et al., 2013, Oddy and Barry, 2009, Pfefferbaum et al., 1985). The N200nogo, an increased negative deflection at fronto-central electrodes in the Nogo condition, has been associated with conflict detection (Donkers and Van Boxtel, 2004, Eimer, 1993, Fallgatter et al., 2002). On the other hand, the P300 component has been correlated with behavioral success while performing executive tasks, and response inhibition (in the Nogo condition) is more cognitively demanding than the response activation (in the Go condition (Bokura et al., 2001, Polich, 2007, Ruchsow et al., 2008). Our previous research reported that pituitary patients showed diminished amplitudes of N200nogo and P300nogo compared to the healthy controls (HCs). These findings implicated the patients’ deficits in response activation and inhibition (Cao et al., 2017).

Existing studies mainly focused on the broad-band Event-related potentials (ERPs) which only reflect the phase-locked neural activity evoked by the stimuli. Event-related neural oscillations at different frequency bands could provide more information. Oscillatory activity at distinct frequencies is associated with a variety of cognitive functions (Klimesch et al., 2007, Pandey et al., 2016). Specifically, neural activity at the theta and alpha frequency bands are related to the cognitive control in the Go/Nogo task (Cavanagh and Shackman, 2015, Ishii et al., 1999, Klimesch et al., 2007, Yamanaka and Yamamoto, 2010). Theta modulations are essential for recruiting cognitive control processes related to response execution and inhibition (Cohen and Donner, 2013). Reports have demonstrated increased theta power in the frontal midline areas during the suppression of non-target trials (Barry, 2009, Yamanaka and Yamamoto, 2010). Moreover, intracranial electroencephalography (iEEG) recordings have found the anterior cingulated cortex (ACC) is a major intracranial structure that generates the theta activity observed in the frontal midline areas (Cohen et al., 2008). Similarly, the importance of alpha oscillation in the active suppression of irrelevant or distracting information has been reported (Cooper et al., 2003, Sadaghiani et al., 2012). Some studies reported that alpha modulation acted as the control mechanism operating via inhibition and robust alpha power would result in inhibiting unwanted or irrelevant information (Foxe and Snyder, 2011, Jensen and Mazaheri, 2010). Specifically, alpha activity is particularly relevant due to their association with cognitive control in Go/NoGo task (Nguyen et al., 2017).

So far, few studies have examined the changes of evoked oscillations corresponding to a visual Go/Nogo paradigm in prolactinoma patients. Previous research used scales (e.g., MiniMental State Examination (MMSE) and Cambridge Cognitive Examination-Chinese version (CAMCOG-C) tests) to identify the factors that affect the cognitive functions of pituitary adenoma patients. Besides the scales, Cao et al. (2017) recruited three subtypes of pituitary adenoma (prolactinomas, growth hormone secreting pituitary tumors, and nonfunctional pituitary tumors) and therefore could not investigate how the PRL hypersecretion affecting the brain activity and further impairing the behavioral performance. Thus, in this study, we aimed to examine changes in the ERPs and neural oscillations in prolactinoma patients to reflect the impaired response activation and response inhibition. Based on the significant link between cognitive controls and theta and alpha bands mentioned above, the present research would mainly focus on the theta-band and alpha-band activity. We hypothesized that the prolactinomas would show the smaller amplitudes of N200 and P300, and more importantly lower theta and alpha power compared to the HCs.

## 2 Methods

### 2.1 Participants

Prolactinoma patients were recruited in the Department of Neurosurgery, Wuhan School of Clinical Medicine, Southern Medical University (China). Inclusion criteria were as follows: (1) Patients were diagnosed with a prolactin-secreting pituitary tumor (Melmed et al., 2011), (2) aged between 20 and 50 years and were above the middle school level (the education time is more than 9 years) with the upper-limit of 15-year education, (3) had no history of craniotomy or radiation therapy, (4) had a normal or corrected-to-normal vision, (5) could complete EEG tests. Patients were excluded if they (1) had a history of neurologic or psychiatric disorders, (2) had comorbidities that could affect cognitive function, for example, severe liver, heart or kidney dysfunction, (3) had severe complications, such as coma, infection, epilepsy, hydrocephalus, and leaking of cerebrospinal fluid, (4) had drug or alcohol abuse [subjects who drink alcohol over 2.0 standard drinks (10 g of pure alcohol) during the day and meet any 2 of the 11 criteria under the DSM-V (Association American Psychiatric, 2013) in the past year, or were on any medications (including oral contraceptives). Considering circadian changes in hormone levels, 20 to 50 years and all the patients were above middle school level (the education time is more than 9 years) with the upper-limit of 15-year education.vein blood samples were collected in the morning between 8:00 and 9:30. Because we only recruited the prolactinomas, this research mainly focused on serum PRL (ng/ml). PRL was diluted to 1:100 in order to avoid the hook effect. Tumor size may have underlying effects on our results because studies have shown the brain structure changes in pituitary patients, which were caused by macroadenomas (Rutland et al., 2020, Rutland et al., 2019). In order to eliminate the mass effect of the tumor on adjacent neuroanatomical structures, the study population was strictly selected to rule out big tumor size that compresses optic nerves or surrounding brain structures.

In total, we tested 25 patients and recorded their scalp EEG while they were performing the task. Two patients’ data set could be loaded due to technical problems and therefore they were removed from the analysis. Two more patients were excluded from statistical analysis because of huge artifacts that couldn’t be removed or corrected through preprocessing. Thus, the final sample was consisted of 21 patients (see Table 1). The HCs were recruited from healthy volunteers with matched age (t (40) = 1.404, *p* = .168), gender (χ2 = 0.104, *p* = .747) and education (t (40) = 0.863, *p* = .393). Similar studies using Go-Nogo task have also reported medium to large effect size with a sample size of 20ish (e.g., Kaiser et al., 2003, Nguyen et al., 2017). The study was approved by the ethics committee of Wuhan School of Clinical Medicine, Southern Medical University. The written informed consent was explained carefully and obtained from all participants.

**Table 1:** Demographic and clinical characteristics of prolactinoma patients and healthy controls (HCs).

		Prolactinoma patients (n = 21)	HCs(n = 21)	*p*-Value
Age (years)		35.1 (30–40)	33.9 (27–37)	0.107^a^
Gender	Male	7	8	χ2 = 0.104, 0.747^b^
	Female	14	13	
Education (years)		12.3 (9–15)	12.9 (9–16)	0.783^a^

Data are presented as mean values and ranges (minimum and maximum values)

a one-way analysis of variance (ANOVA),

b Chi-Square Tests.

### 2.2 Stimuli and procedure

**Fig. 1:**
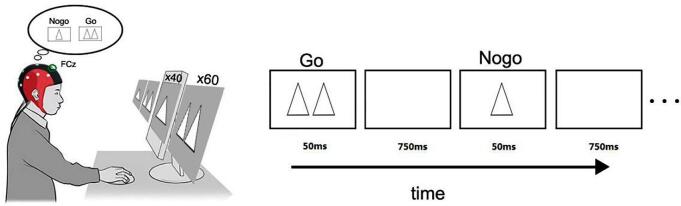
The procedure of Go/NoGo task. Detailed electrodes such as electro-oculograms are omitted in this figure. The Go stimuli for targets are double triangles (n = 60 per block), and the Nogo stimuli for nontargets are Single triangles (n = 40 per block) with pseudorandom order. The interstimulus interval was between 700 ~ 800 ms with a mean of 750 ms. There were 3 blocks in total.

Participants sat in the semi-dark test room with the screen 100 cm away from the eyes. Double-triangle was applied as target stimuli (Go) and single-triangle was applied as nontarget stimuli (Nogo). These stimuli appeared on the central computer screen (physical luminance = 60 cd/m^2^). In each trial, the stimulus lasted 50 ms, followed by the interstimulus interval being 750 ms (randomly between 700 and 800 ms). Once the Go stimuli appeared on the screen, the participant should quickly press the button, but do not press the button when the Nogo stimuli appeared. There were three blocks in total, each of which contained 60 Go and 40 Nogo trials (see Fig. 1). Participants made a full practice before the formal EEG recording. During the whole process, participants needed to watch the screen center leisurely and reduce their eye blinks and body movements as possible as they could.

### 2.3 EEG recording:

The EEG was acquired by a 64-channel array with the international 10–20 system (eegoTM amplifier, Germany) linked to both earlobe reference electrodes built-in an elastic cap. The impedance levels of EEG recording were under 5 KΩ. EEG signals were continuously recorded with a band pass of 0.05–200 Hz. The sampling rate was at 1000 HZ during acquisition.

### 2.4 Data analysis

Behavior. We calculated hit rate and false alarm based on participants’ responses in Go and Nogo trials. Following the signal detection theory, detection sensitivity (d’) was computed to measure the response inhibition (d’ = Z_hit rate_– Z_false alarm_, Z(p), p ∈ [0,1], is the inverse of the cumulative distribution function of the Gaussian distribution). Standard corrections were performed to deal with hit rate of 1 or false alarm rate of 0 (Macmillan and Kaplan, 1985). Mean reaction time (RT) was obtained from the correct Go trials. Between-group permutation tests for difference in means (two-tail, alpha = 0.05, permutation times = 10000) were performed on RT, hit rate and false alarm to explore the group difference between the prolactinoma patients and the HCs.

Preprocessing. Offline preprocessing was performed via EEGLAB (Delorme and Makeig, 2004). We bandpass-filtered the data to 1–40 Hz and re-referenced the data to the averaged mastoids. Bad channels were spherically interpolated (One participant’s FT electrode was interpolated). Independent Component Analysis was performed to correct ocular movements and other artifacts. In case that there were unexpected artifacts, we set a threshold of 50uV to exclude trials where any electrode still had abnormal amplitudes beyond the threshold at any time point after preprocessing. On average, there were 8.4%+6.6% trials being discarded for the following analysis.

Event-related potential (ERP) analysis: Custom scripts were used for the ERP analysis. The continuous EEG was segmented into the epoch from 0.2 s before stimulus to 0.7 s after the stimulus, and baseline-corrected to the mean amplitude of pre-stimulus interval. Epochs in which any channel contaminated by artifacts exceeding amplitude of ± 50 μV were removed from averaging. After this procedure, the EEG segments were averaged separately for Go and Nogo stimuli. FCz and Pz were selected as the target channels representing the frontal and parietal lobe (e.g., Kaiser et al., 2003, Kiefer et al., 1998). We performed mixed three-way ANOVA on the averaged amplitudes between 0.35 ~ 0.45 s for the P300 component. The between-subject factor was group (HCs/Patients) and the two within-subject factors were conditions (Go/Nogo) and electrodes (FCz/Pz). To reveal patients’ deficits in inhibitory control, we performed the non-parametric permutation test (iteration = 10000) to compare the group difference on the averaged amplitude of N200nogo between 0.23 ~ 0.33 s. We permuted the group labels for 10,000 times and obtained the distribution of the permuted difference. If the original mean difference ranks higher than 5% among the permuted mean differences, we reject the null hypothesis and conclude that the observed mean difference didn’t occur by chance.

Time-frequency analysis: We performed time–frequency decomposition using Fieldtrip (Oostenveld et al., 2011) and customized Matlab scripts (MathWorks, Natick, MA, USA). Epochs were selected between −3s and 3 s relative to stimulus onset. The extra time points were added to avoid the edge effect (Cohen, 2014). After the time–frequency decomposition, we selected data between −0.4 s and 0.8 s for further statistical testing. The surface Laplacian filtering was performed to decrease the volume conduction. Morlet wavelet convolution with a kernel width of 7 was used to extract the time–frequency activity every 50 ms. The frequencies ranged from 1 ~ 30 Hz in 30 linearly spaced steps. We averaged power values across trials for each condition and divided by the mean of pre-stimulus baseline (-400 ~ -100 ms). Cluster-based permutation was performed on the time–frequency data over participants. Adjacent time–frequency time points exceeding the threshold (alpha = 0.0001, two-tail) were grouped as a cluster. The cluster-level statistic was calculated by taking the sum of the difference values within the cluster. The number of random permutations using the Monte Carlo method was set to 10000. Theta oscillation at the mid-frontal cortex (MFC) has been regarded as an essential neural activity related to cognitive control (Cavanagh and Frank, 2014). EEG signal at the FCz electrode dominantly reflects the neural activity of the MFC (Nigbur et al., 2011). Previous studies also selected parietal-occipital electrodes, particularly PO7, PO8, and Pz as the scalp regions of interest (Piispala et al., 2018). To further reveal the oscillatory difference between patients and the healthy controls, we extracted the theta band (3–7 Hz) power at the mid-frontal cortex (FCz) and alpha band (8–12 Hz) power at the parietal cortex (PO7 and PO8) using Hilbert transform for each condition and normalized to the averaged power of baseline period. Compared to the wavelet convolution, Hilbert transform allows more control over the frequency characteristics (Cohen, 2014). Hence, once identified the frequency band of interest through whole-brain time–frequency analysis, we further investigated the difference between the two groups at the specific frequency band. The time period of 200 ~ 400 ms was used for theta-band activity based on previous studies while 0 ~ 300 ms was used for the alpha-band activity. Mixed ANOVA with group (HCs/Patients) being the between-subject fact and condition (Go/Nogo) being the within-subject factor was performed to examine the averaged power difference of the selected time interval at the corresponding electrode site.

Mediator model: We conducted mediation analyses (Hayes, 2017) to examine the relationship among PRL level, theta oscillation and behavior. Two mediation models were reported depending on which behavioral index was used as the representation of task performance. In the first model, we used d’, which represents the response sensitivity in Go and Nogo condition, to reflect participants’ response inhibition ability in the current task. Correspondingly, the theta activity was measured as the power difference at the theta band between Nogo and Go condition during 0.2 ~ 0.4 s, representing the response inhibition (Harper et al., 2018). In the second model, we took the false alarm rate as the reflection of inhibitory control and therefore set theta power in the Nogo condition as the predictor. The PRL level was added as the mediator and the indirect effect was tested using non-parametric bootstrapping with 10,000 iterations. The 95% confidence interval was computed by determining the indirect effects at the 2.5th and 97.5th percentiles.

## 3 Results

• Behavior

**Fig. 2:**
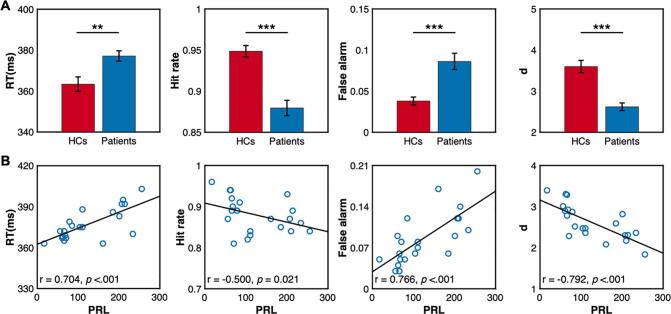
Behavioral performance of the HCs and the patients. A) Group comparisons between the patients and the HCs. The errorbar represented the between-subject standard error. * *p* < .05, ** *p* < .01, *** *p* < .001 (two-tailed). B). Correlations between PRL and behavioral performance of the patients.

Patients’ behavioral performance was generally worse than the HCs in both Go and Nogo trials. They showed slower RT and lower hit rate in Go trials compared to the HCs (RT: t(40) = 3.164, *p* = .002, Cohen’s d = 0.691; Hit: t(40) = 5.930, *p* < .001, Cohen’s d = 1.294). Besides, patients’ false alarm was higher than the HCs in the Nogo trials (t(40) = 4.372, *p* < .001, Cohen’s d = 0.954). The d’ was higher in the HCs than the patients (t(40) = 5.586, *p* < .001, Cohen’s d = 1.219). Taken together, these results demonstrated that patients’ response control ability in execution and inhibition was deteriorated (see Fig. 2A).

**Fig. 3:**
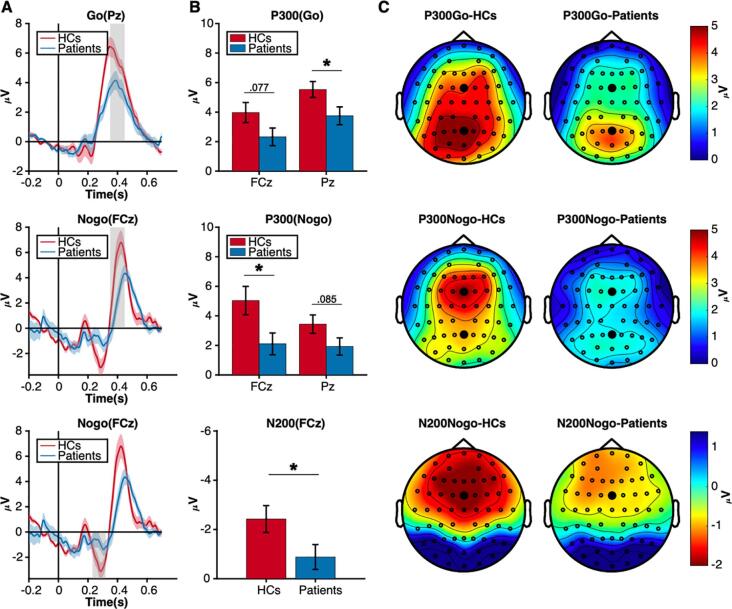
P300 and N200 of the HCs and the patients. A). Waveforms of P300 and N200. P300 of Go (top panel) and Nogo (middle panel) conditions were calculated from Pz and FCz respectively. N200nogo was found at the FCz (bottom panel). The shaded errorbar was the between-subject standard error. Gray rectangles index time windows for ERPs amplitudes analysis. B). Group difference of P300 and N200. The bar represented the P300 or N200 amplitudes of the gray rectangle in Fig. 3A. The errorbar represented the between-subject standard error. * *p* < .05, ** *p* < .01, *** *p* < .001 (two-tailed). C). The topography of P300 and N200 components.

To reveal how hypersecretion of PRL affects patients’ cognitive functions at the individual level, Spearman's Rank correlation coefficients between PRL and behavior were measured. RT and false alarm rate were positively correlated with the PRL (RT, r = 0.704, *p* < .001; false alarm, r = 0.766, *p* < .001). Patients with overproduction of PRL had lower hit rate (r = -0.500, *p* = .021) and smaller d’ (r = -0.792, *p* < .001).

• Decreased amplitudes of P300 and N200 in prolactinomas

As for P300 component, we observed a significant three-way interaction effect (group × condition × electrode: F(1,40) = 7.142, *p* = .011, η_p_^2^ = 0.151). There were significant main effect for group (F(1,40) = 5.954, *p* = .019, η_p_^2^ = 0.130) and condition (F(1,40) = 4.699, *p* = .036, η_p_^2^ = 0.105), but not for electrodes (F(1,40) = 0.757, *p* = .390, η_p_^2^ = 0.019). Two-way interaction effect for condition and electrode was significant (F(1,40) = 66.925, *p* < .001, η_p_^2^ = 0.626) but not for condition and group (F(1,40) = 0.513, *p* = .478, η_p_^2^ = 0.013) nor electrodes and group (F(1,40) = 0.854, *p* = .361, η_p_^2^ = 0.021). We further examined the three-way interaction by investigating the Go and Nogo condition separately.

In the Go condition, we found significant main effect for group (F(1,40) = 4.91, *p* = .032 , η_p_^2^ = 0.109) and electrodes(F(1,40) = 16.20, *p* = .001 , η_p_^2^ = 0.288), and their interaction effect was not significant (F(1,40) = 0.03, *p* = .856, η_p_^2^ = 0.001). These indicated that the patients had an overall smaller P300nogo than the HCs and the P300 component was stronger in the parieto-occipital region. In the Nogo condition, there were significant main effect for group (F(1,40) = 5.17, *p* = .028 , η_p_^2^ = 0.114) and electrodes (F(1,40) = 5.39, *p* = .025 , η_p_^2^ = 0.119) and their interaction effect was marginally significant (F(1,40) = 3.44, *p* = .071 , η_p_^2^ = 0.079). Simple simple effect analysis indicated that the HCs’ P300 was larger than the Patients only at the FCz (F(1,40) = 5.85, *p* = .020 , η_p_^2^ = 0.128) but not at the Pz (F(1,40) = 3.12, *p* = .085, η_p_^2^ = 0.072). As for the N200nogo, the HCs had a stronger Nogo effect relative to the patients (t(40) = 2.074, *p* = .049, Cohen’s d = 0.442).

• Whole-brain time–frequency analysis

**Fig. 4:**
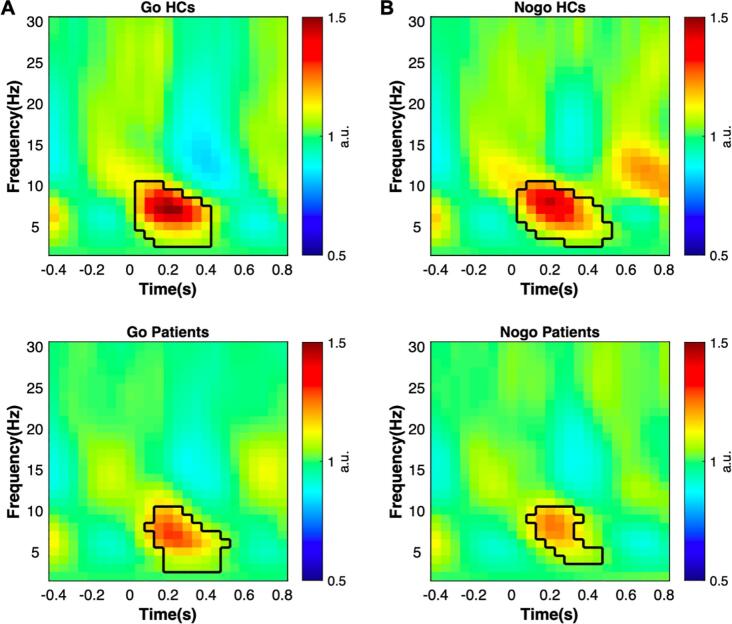
Neural oscillations evoked by the stimuli in Go and Nogo conditions. A) Significant clusters at the theta and alpha band in Go condition. HCs, 3–10 Hz, 0.05–0.4 s, *p* < .001; Patients, 3–10 Hz, 0.1–0.5 s, *p* < .001. Y-axis starts from 2 Hz. B) Significant clusters at the theta and alpha band in Nogo condition. HCs, 3–10 Hz, 0.05–0.5 s, *p* < .001; Patients, 4–10 Hz, 0.10 ~ 0.45 s, *p* < .001.

Whole-brain time–frequency analysis was performed to identify the stimulus-evoked neural oscillations. The results showed that significant oscillatory activity between 3 ~ 10 Hz was evoked in the HCs and the patients (see Fig. 4 for details). To further investigate the evoked oscillatory dynamics of the two populations, we separately extracted theta-band (3–7 Hz) and alpha-band (8–12 Hz) power at the FCz and Pz.

• Lower occipital alpha power in prolactinomas

Alpha-band activity in the Go and Nogo conditions showed a parieto-occipital distribution. Mixed ANOVA showed that there was a marginal significance of group (F(1,40) = 3.749, *p* = .060, η_p_^2^ = 0.086). Patients had lower occipital alpha power than the HCs, indicating a damaged early attention control. Main effect of condition (GO/Nogo) was not significant (F(1,40) = 0.055, *p* = .816, η_p_^2^ = 0.001) either was the interaction between group and condition (F(1,40) = 0.023, *p* = .880, η_p_^2^ = 0.001).

• Lower frontal theta power in the prolactinomas

**Fig. 5:**
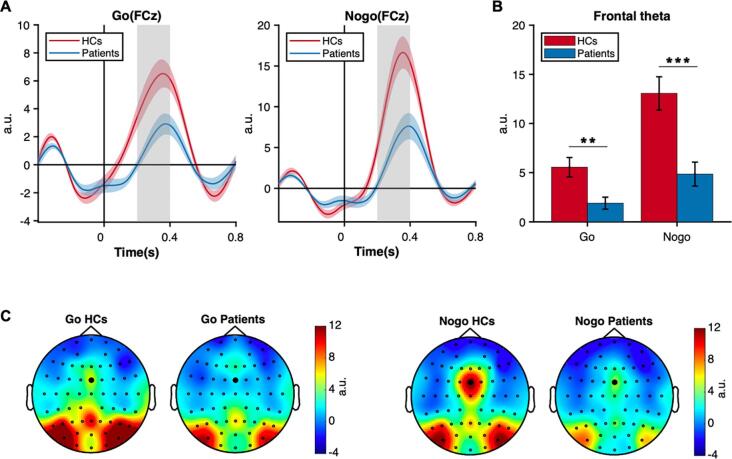
Frontal theta oscillation. A). Time series of theta power at FCz in Go and Nogo condition. The gray rectangle indicated the time interval of interest. B). Group comparisons of average theta power between grey rectangle in Fig. 5A. * *p* < .05, ** *p* < .01, *** *p* < .001 (two-tailed). C) The topography of the average theta power.

**Fig. 6:**
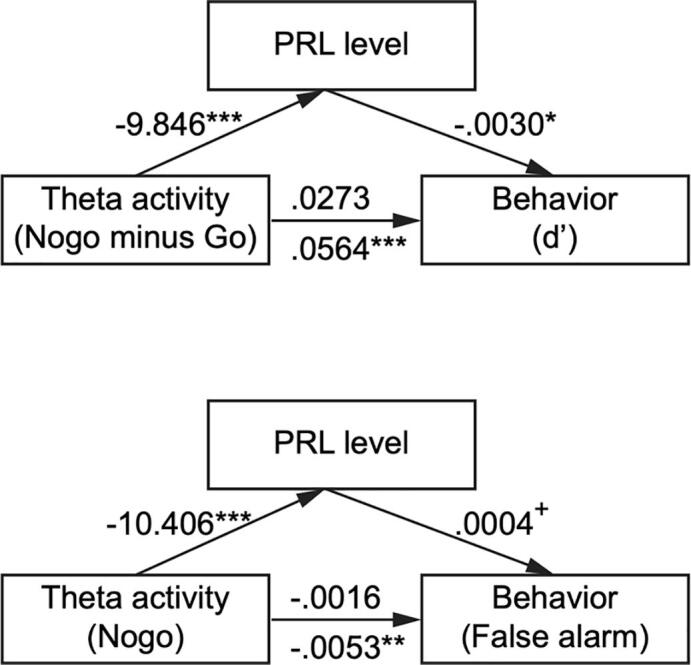
Mediator model. PRL hypersecretion mediated the influence of MFC theta power on behavior. The first model examined the relationship between theta power (Nogo minus Go) and d’ while the second model focused on false alarm and theta power in the Nogo condition. ^+^ represented marginal significance (*p* = .052). The number above path *c* (from theta activity to behavior) was the direct effect and the number below was the total effect.

As showed in Fig. 5, the HCs had overall stronger frontal theta power than the patients (F(1,40) = 16.623, *p* < .001, η_p_^2^ = 0.294) and theta power in the Nogo condition was higher than the Go condition (F(1,40) = 37.049, *p* < .001, η_p_^2^ = 0.481). However, their interaction was also significant (group × condition: F(1,40) = 7.003, *p* = .012, η_p_^2^ = 0.149). Simple effect analysis showed that patients had generally smaller theta power in the Go condition (F(1,40) = 9.98, *p* = .003, η_p_^2^ = . 200) and the Nogo condition (F(1,40) = 15.41, *p* < .001, η_p_^2^ = 0.278).Fig. 6.

To further investigate the cognitive function of theta and its behavioral impact, we calculated Spearman’ Rank correlation coefficients between theta power and behavioral indices in the HCs group. Significant negative correlation was found between theta power in the Go trials and participants’ RT (r = -0.799, *p* < .001) while a positive correlation was found with hit rate (r = 0.682, *p* < .001). Hence, stronger theta activity in the Go condition was related to better performance. As for the Nogo condition, participants who had stronger theta activity would be less prone to false alarm (r = -0.898, *p* < .001). As for the patients, the correlations between frontal theta and behavioral indices showed coherent trends but were not as typical as the HCs (Go theta & RT, r = -0.332, *p* = .141; Go theta & hit rate, r = 0.458, *p* = .037; Nogo theta & false alarm, r = -0.758, *p* < .001).

• PRL hypersecretion mediated the correlation between behavior and frontal theta oscillation

We conducted mediation analysis to showed the relationship between frontal theta activity and behavior performance. In the first model, the path from theta activity to behavior (d’) was significantly positive (total effect, *b* = 0.0564, t(19) = 4.048, *p* < .001). Moreover, the effect of theta activity on PRL (*b* = -9.846, t(19) = -4.247, *p* < .001) and the effect of PRL on behavior was also significant (*b* = -0.003, t(18) = -2.398, *p* = .028). The direct effect of theta power on d’ (*b* = -0.0016, t(18) = -0.731, *p* = .474) was no longer significant once adding PRL as the mediator. Thus, the mediation model was valid and the indirect effect of PRL was 0.0291 (CI = [-0.0036 0.0593]). Similarly, the path from theta activity to behavior (false alarm) in the second model was significantly positive (total effect, *b* = -0.0053, t(19) = -3.773, *p* = .001). The effect of theta activity on PRL was significant (*b* = -10.406, t(19) = -5.973, *p* < .001) but the effect of PRL on behavior was not significant (*b* = 0.0004, t(18) = 2.079, *p* = .052). However, the bootstrapped unstandardized indirect effect -0.0037 was significantly different from 0 (CI = [-0.0074, -0.0006]), suggesting that the mediation model was valid too. Taken together, these results indicated that PRL was a mediator of theta activity and behavior.

## 4 Discussion

The current study examined ERPs and oscillatory differences between the prolactinoma patients and HCs related to response activation and response inhibition using a Go/Nogo task. Prolactinoma patients showed longer reaction time, lower hit rate, higher false alarm and detection sensitivity (d’) than the HCs, indicating a deteriorated response control ability. Importantly, PRL levels correlated with behavioral results (including RT, hit rate, false alarm and detection sensitivity). Compared to the HCs, patients had lower frontal P300nogo and lower parietal P300go. Meanwhile, the N200nogo was smaller in the patients. As for neural oscillation differences, patients displayed lower evoked frontal theta power in the Nogo and Go conditions compared to the HCs, implying a worse response control ability. Furthermore, the mediator model suggested that the relationship between frontal theta power and inhibitory ability was mediated by abnormally high PRL levels. Additionally, patients showed lower occipital alpha power in both conditions, indicating deficits in both response activation and inhibition of neural circuits underlying the required behavior.

Prolactinomas showed decreased amplitudes of P300 in both the Go and Nogo conditions. P300go component is correlated with behavioral success in performing executive tasks whereas P300nogo is associated with response inhibition (Gao et al., 2017, Ruchsow et al., 2008). The decreased P300go implied prolactinomas’ deficits in response activation. Moreover, prolactinomas patients also manifested smaller P300nogo. P300nogo modulation is generally considered to be an inhibitory mechanism. P300 amplitudes decline has been reported in various studies investigating the cognitive dysfunctions of response control among psychological and psychiatric patients (Fallgatter et al., 2003, Kleinlogel et al., 2007). In support of previous literature, we herein observed significant P300nogo decrease on prolactinomas patients at the frontal region and in the Go condition at the parietal region. These findings proved our hypothesis that these patients’ ability to execute and inhibit the response was impaired. Topographically, the P300go is most active at posterior regions (including centro-parietal electrodes), whereas the P300nogo is primarily active at anterior regions including fronto-central electrodes (Kaiser et al., 2003, Smith et al., 2006). Our study also proved the Nogo anteriorization regardless of the group (Fallgatter and Strik, 1999). Furthermore, N200nogo has also been characterized as an index of conflict inhibition or conflict monitoring (Donkers and Van Boxtel, 2004, Fallgatter et al., 2002). The decreased N200nogo in the prolactinomas relative to the HCs indicated that N200 may be one of the measures related to the impaired inhibitory control.

Prolactinomas also showed lower frontal theta power in both the Go and Nogo conditions. Considering the relationship between theta oscillation and cognitive functions (Cavanagh and Shackman, 2015, Pandey et al., 2016, Yamanaka and Yamamoto, 2010), these results indicated that patients’ deficits in executing and inhibiting the response in the Go and Nogo conditions respectively. Our previous research found that GMV of prolactinoma patients decreased, especially in the orbitofrontal cortex and frontal cortex (Yao et al., 2018). This provided a possible explanation why prolactinomas showed lower frontal theta power. Importantly, theta power in the Go condition positively correlated with hit rate but negatively correlated with RT, indicating that frontal theta power decrease would result in execution disability. Moreover, MFC theta is associated with response inhibition as evidenced by the negative correlation between theta power and false alarm in the Nogo condition. The mediation analysis further showed that PRL mediated the relationship between frontal theta power and inhibitory behavior (d’ and false alarm). Taken together, the theta oscillation can be regarded as an index to reflect that PRL overproduction leads to the impaired inhibitory control in prolactinomas. Therefore, the attenuation of theta power can be treated as an early objective electrophysiological marker for prolactinomas.

Our mediation model showed that PRL overproduction mediated the influence of MFC theta power on behaviour. PRL is widely expressed in the brain, including the thalamus, cerebral cortex, hypothalamic, amygdala, etc (Cabrera-Reyes et al., 2017). PRL overproduction will impair cognitive abilities (Torner et al., 2013). Bala et al. (2016) speculated that the PRL overproduction might influence the efficiency of cognitive functioning via the dopamine pathway, which had been altered in prolactinoma patients because of the anti-correlation between dopamine secreting and prolactin production (Ben-Jonathan and Hnasko, 2001). Biologically, PRL overproduction is relevant in neuronal changes and plasticity in the brain cortex because PRL significantly enhances the number of cells secreting antibodies directed against myelin oligodendrocyte glycoprotein (Correale et al., 2014). Oligodendrocytes are a type of neuroglia whose main functions are providing support and insulation to axons in the central nervous system of some vertebrates (Ragheb, 1999). Hence, abnormally high PRL levels are detrimental to cerebral gray matter, then resulting in the lower theta power and poor inhibitory control.

Prolactinomas had lower occipital alpha band power in both the Go and Nogo conditions compared to the HCs. Occipital alpha power increase has been associated with the inhibition of unnecessary or distracting visual information (Foxe and Snyder, 2011) and with reduced cortical excitability (Romei et al., 2008). Related theories have suggested that the increase in alpha power may reflect a suppression of irrelevant posterior areas (Başar, 2006, Klimesch et al., 2007). Based on this interpretation, Tuladhar et al. (2007) found that alpha amplitudes reflected the suppression of unnecessary visual processes to support effective cognitive processes. The idea is that effective cognition is a function of how efficiently the brain works rather than how hard it works (Klimesch et al., 2007). In our study, the occipital alpha in the HCs during the Go/Nogo task represented efficient disengagement of visual perception, which is in line with other studies that the occipital alpha synchronization reflects suppression of the unnecessary visual stream (Cooper et al., 2003, Tuladhar et al., 2007). In contrast, prolactinomas manifested lower occipital alpha power, which indicates their dysfunction of inhibiting non-essential visual information and preventing interfering signals from affecting the effective cognitive processes.

## 5 Limitations

Several limitations should be addressed. First, we recorded the electrophysiological signals at the scalp level and therefore we cannot point out which neuroanatomical regions exactly are dysfunctional in prolactinomas. Second, conjunct analyses integrating various neuroimaging methods, such as structure MRI and fMRI (Catalino et al., 2020), should be conducted to map a more comprehensive cognitive and emotional network as well as detect both hemodynamic and electrical sources of neural activity in the future. Third, since big tumors were excluded by qualitative evaluation, future studies should better quantify the tumor size via MRI evaluation.

## 6 Conclusion

Taken together, both ERPs and oscillatory differences were observed between the prolactinoma patients and HCs related to response activation and inhibition. Patients showed impaired response activation as reflected by the smaller parietal P300go and deteriorated response inhibition as showed by the lower frontal P300nogo and N200nogo. We also observed lower frontal theta activity and occipital alpha activity in both conditions. It can thus be concluded that prolactinoma patients show cognitive deficits in both executive function and inhibitory control. The dysfunctions of execution and inhibition may be attributed to the hypersecretion of PRL. PRL levels play a pivotal role in mediating the correlation between frontal theta oscillation and behavioral performance. Therefore, the frontal theta oscillation could be one of the potential biomarkers that predict the inhibitory dysfunction in prolactinomas.

Ethics statement

All procedures followed the Declaration of Helsinki and were approved by the Ethical Committee of Wuhan School of Clinical Medicine, Southern Medical University (China). The number of the approved ethical statement is “[2014] 024–1.” All subjects were fully informed of the nature of the study protocol and all gave their written consent regarding participation.

## CRediT authorship contribution statement

Guozheng Xu and Jian Song were responsible for the conceptualization and funding acquisition. Chenglong Cao, Pan Ma and Binbin Liu were responsible for the data curation. Chenglong Cao and Wen Wen assisted with the formal analysis, investigation and methodology. Chenglong Cao and Wen Wen completed the original draft and review the draft. Chenglong Cao and Wen Wen contributed to the project administration. Sheng Li critically supervised the original draft. All authors have reviewed the content and approved the final version for publication.

## Declaration of Competing Interest

The authors declare that they have no known competing financial interests or personal relationships that could have appeared to influence the work reported in this paper.
